# Digital Divide or Educational Divide? Impact of Social Media on Covid-19 Vaccination in Older Adults

**DOI:** 10.1093/geroni/igaf122.3398

**Published:** 2025-12-31

**Authors:** Mason Dupre, Radha Dhingra, Truls Østbye, Teah Bayless, Hanzhang Xu

**Affiliations:** Cary Academy, Cary, North Carolina, United States; Duke University School of Medicine, Durham, North Carolina, United States; Duke University School of Medicine, Durham, North Carolina, United States; Duke University School of Medicine, Durham, North Carolina, United States; Duke University, Durham, North Carolina, United States

## Abstract

The spread of disinformation and harmful content on social media is well-known. However, most research focuses on the negative influence of social media on the well-being of adolescents and teenagers. We used data from the 2022 Health and Retirement Study (n = 4,038) to examine how the use of social media (Facebook, Twitter [X], and Instagram) was associated with vaccination uptake for COVID-19 among U.S. middle-aged and older adults. Frequency of social media use was measured on a scale ranging from 0=never to 5=daily. In our study (mean (SD) age: 69.4 (9.7) years), approximately 36% of participants used social media daily while 34% never did. Frequent media users were more likely to be younger, women, and more educated compared with less frequent/non-users of social media. Logistic regression models showed that social media use was not directly associated with Covid-19 vaccination. However, we found a significant interaction effect that vaccination uptake for COVID-19 was significantly lower in older adults with low education who frequently used social media. Among adults who used social media daily, those with less than a high school education were significantly less likely to get vaccinated than those with a college degree (odds ratio [95% CI]: 0.35 [0.15-0.82]; P=.016). These results remained after accounting for differences in participants’ sociodemographic background, health status, religiosity, and other factors. Furthermore, social media use was not associated with vaccination uptake for influenza, pneumonia, or shingles. Our results highlight the importance of delivering tailored public health messages when promoting vaccine uptake among less-educated older populations.

